# Evaluating different web applications to assess the toxicity of plasticizers

**DOI:** 10.1038/s41598-022-18327-0

**Published:** 2022-11-16

**Authors:** Charli Deepak Arulanandam, Jiang-Shiou Hwang, Arthur James Rathinam, Hans-Uwe Dahms

**Affiliations:** 1 Department of Biomedical Science and Environmental Biology, Kaohsiung Medical University, Kaohsiung, 80708 Taiwan, ROC; 2 Department of Medicinal and Applied Chemistry, Kaohsiung Medical University, Kaohsiung, 80708 Taiwan, ROC; 3 Institute of Marine Biology, National Taiwan Ocean University, Keelung, 20224 Taiwan, ROC; 4 Center of Excellence for Ocean Engineering, National Taiwan Ocean University, Keelung, 20224 Taiwan, ROC; 5 Center of Excellence for the Oceans, National Taiwan Ocean University, Keelung, 20224 Taiwan, ROC; 6 Department of Marine Science, Bharathidasan University, Tiruchirappalli, 620 024, India; 7 Research Center of Precision Environmental Medicine, Kaohsiung Medical University, Kaohsiung, 807 Taiwan; 8 Department of Marine Biotechnology and Resources, National Sun Yat-Sen University, No. 70, Lienhai Road, Kaohsiung, 80424 Taiwan, ROC

**Keywords:** Biotechnology, Computational biology and bioinformatics

## Abstract

Plasticizers increase the flexibility of plastics. As environmental leachates they lead to increased water and soil pollution, as well as to serious harm to human health. This study was set out to explore various web applications to predict the toxicological properties of plasticizers. Web-based tools (e.g., BOILED-Egg, LAZAR, PROTOX-II, CarcinoPred-EL) and VEGA were accessed via an 5th–10th generation computer in order to obtain toxicological predictions. Based on the LAZAR mutagenicity assessment was only bisphenol F predicted as mutagenic. The BBP and DBP in RF; DEHP in RF and XGBoost; DNOP in RF and XGBoost models were predicted as carcinogenic in the CarcinoPred-EL web application. From the bee predictive model (KNN/IRFMN) BPF, di-n-propyl phthalate, diallyl phthalate, dibutyl phthalate, and diisohexyl phthalate were predicted as strong bee toxicants. Acute toxicity for fish using the model Sarpy/IRFMN predicted 19 plasticizers as strong toxicants with LC50 values of less than 1 mg/L. This study also considered plasticizer effects on gastrointestinal absorption and other toxicological endpoints.

## Introduction

In everyday life, plastics are utilized for various applications and provide a variety of benefits to consumers, including medical treatment, and technological breakthroughs^1^. At the same time, plasticizers are emerging contaminants of food products from a variety of food-packaging. They cause health risks to organisms and humans^2,3^. Environmental concerns include the build-up of waste in landfills, as well as contaminations of ground, coastal and increasingly also open ocean waters. Chemical compounds which are used in soft, flexible, and transparent plastic production can contaminate aquatic and marine environments if they are soluble and hydrophilic. Volatile plasticizers may cause atmospheric pollution and affect the respiration in human and other organisms. Plasticizers as emerging contaminants were detected in environmental partitions of the geo-, atmo-, hydro- and bio-sphere, such as in dust, air, wastewater, drinking water, organisms and food^4^. Plasticizers are extensively disseminated into the sea, posing toxic effects to marine organisms^5^ and were found in aerosols above the East China Sea due to fossil fuel burning^6^. Plasticizers can cause adverse effects on the human endocrine systems by consumption of microplastics from various food sources that are contaminated with plasticizers. Plasticizers may readily be absorbed and dispersed in lipids if they are lipophilic, resulting in body malfunctions, age-related bioaccumulation, and bio-magnification throughout trophic webs^7^.

Bisphenols (Bishydroxy-aryl alkanes) are phenol derivatives. BPA alone exceeds 3.8 million tons of production^8,9^. It may be found in a variety of daily items such as water pipelines, computer parts, toys, and thermal papers. There is rising evidence that BPA is detrimental for human health^8,10^. It also has oxidative and mutagenic potential with the ability to cause DNA methylation and oxidative stress^11^. BPA is further carcinogenic, mutagenic, immunotoxic and hepatotoxic. Besides, BPA is suggested to be neurotoxic and teratogenic, however, with inconsistent evidence as yet^12^. BPA exposure enhances the risk of heart diseases, obesity, and diabetes in humans^13^. BPA poses a significant hazard to potable water and food safety^14^. The above examples resulted in substantial research into exposure-associated health issues related to phthalates and plasticizers generally in humans. During lactation, maternal oral exposure to BPA was increasing the probability of mammary carcinogenesis in a dimethylbenzanthracene (DMBA)-induced rat model^6^. The pharmacological effect of BPA in breast cancer cells is mediated through G protein-coupled estrogen receptor 1 (GPER). As a result, GPER-mediated signaling is considered as one of the transduction pathways via which BPA may promote cancer development^15^. BPA was demonstrated to impact the mammary gland, brain, and reproductive system in rat after fetal and postnatal exposure. It also promotes the growth of hormone-dependent cancers^16^.

**Figure 1:**
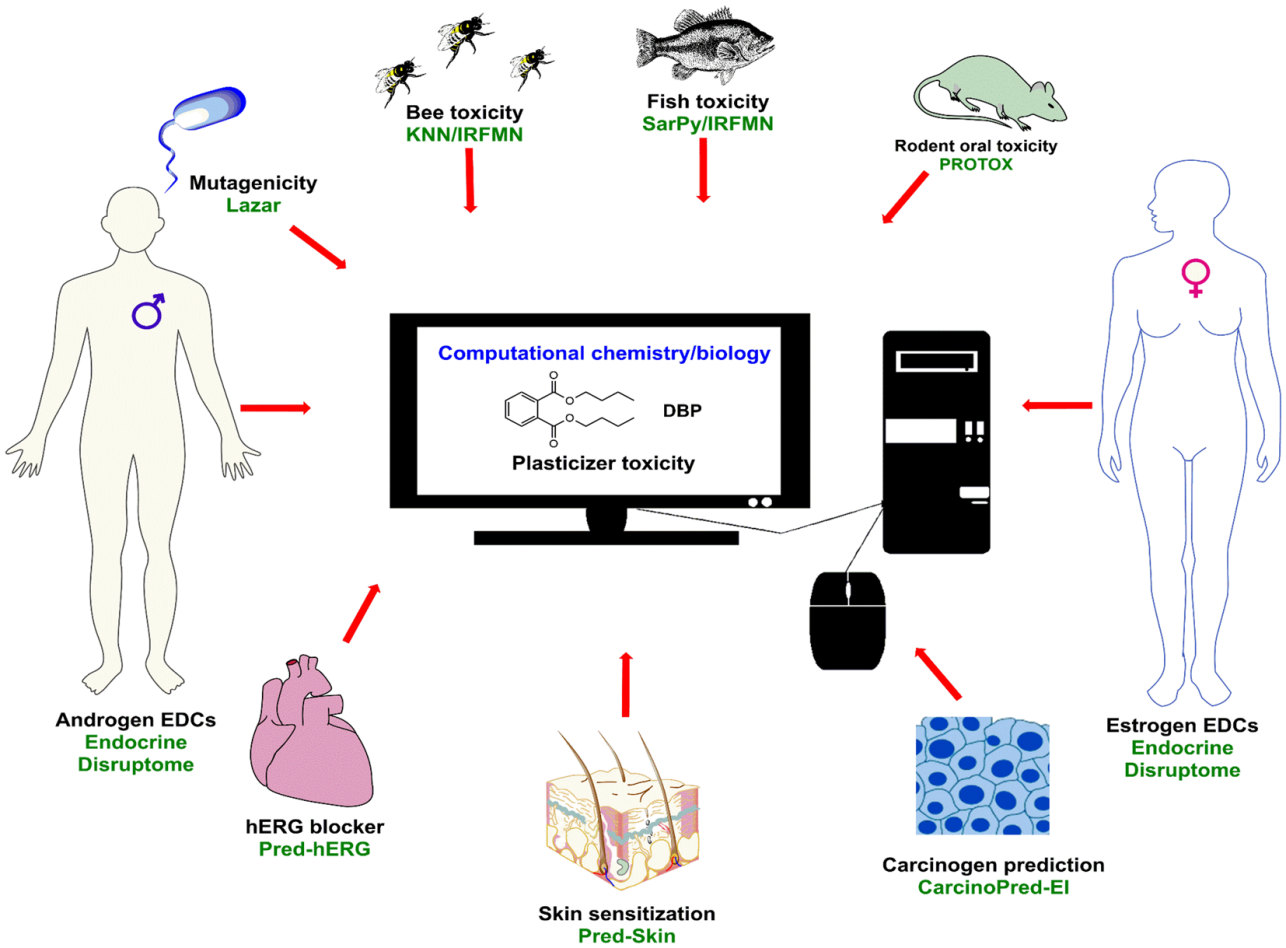
Pictorial representation of in silico analysis of various predictive toxicity analysis (ChemBioDraw Ultra 14—https://scistore.cambridgesoft.com/chembiodraw/).

Followed by customer complaints, BPA was removed from numerous plastic items, resulting in the development and usage of bisphenol alternatives with equally toxic outcomes^17^. In 2000, Fiege and co-workers reported the potential toxicities of bisphenol A derivatives^8^. This holds particularly for analogues such as bisphenol F (BPF) and bisphenol S (BPS). BPF is used to make electrical insulators, epoxy resins, and food packages^5^. Both analogues, BPS and BPF were detected in consumer products of daily use such as paper, and also in products from the food industry^18,19^. Non-toxic or less hazardous chemicals should replace toxic compounds to minimize negative impacts on the environment and the public, according to government laws. Several chemical substitutes, on the other hand, are still untested before being sold and might be hazardous or even more damaging than the original compounds^20^. This also holds for example for perfluorinated compounds used as pesticides^21^. The flame-retardants BPS and BPF, being structural analogues of BPA, may have similar physiological effects compared to BPA. In situ and in vitro screening of compounds for toxicological effects is a costly process in terms of material, infrastructure and human resources^22^. In this situation web applications can provide inexpensive alternatives to predict the toxicological properties of test chemicals (Fig. 1)^23^. These methods can also offer priority criteria for toxicological assessments that should later be tested in vitro. Animal testing will be reduced and replaced, which is both costly and ethically difficult^24^. The use of in silico techniques like QSAR would minimize the use of animals in chemical toxicity testing and improve the efficacy of pollutant risk evaluation for human and environmental health^25^. An important toxicological endpoint here is mutagenicity. Since several mutations are induced by chemicals, it is worthwhile to develop alternative screening methods, such as robust in silico methods to predict chemically induced mutagenicity^26^. In this study, we used the VEGA platform to predict fish and honey bee acute toxicity. We evaluated plasticizer toxicological endpoints and demonstrated applicability and acceptability of web tools and the VEGA software platform.

## Materials and methods

### Data collection of plasticizer chemical structures

Test compound names and abbreviations were retrieved from ChemSpider (S.Table 1). Two-dimensional molecular plasticizer structures were designed using Marvin 17.21.0, ChemAxon and are shown in (S.Fig. 1) based on the available chemical data from ChemSpider^27^.

### SMILES mediated input for the test compounds

Structures of the test molecules are presented as a simplified molecular-input line entry system (SMILES) which were used for toxicity predictions. These SMILES-based assessments were shown to be computationally more efficient in in silico studies^28^. All the test compounds used in this study are shown in S.Table 2 in SMILES format.

## Predictive toxicology

### Mutagenicity prediction

To assess the mutagenicity of chemical compounds, the bacterial reverse mutation assay (AMES test) is frequently applied^29^. To reduce experimental time and materials, the LAZAR web server was used to predict the mutagenicity of plasticizers. This web server was developed and published by Maunz and co-workers in 2013^30^ from available experimental data sets of a bacterial AMES mutagenicity test. The LAZAR toxicity prediction tool is available from https://nano-lazar.in-silico.ch/predict. It provides toxicological predictions by the analysis of compound structures. This holds for mutagenicity, blood–brain barrier penetration, rodent carcinogenicity, and maximum recommended doses. This web server applies for the prediction of toxicity of known and unknown chemical structures^31^. It provides a modular program for toxicity prediction^30^. LAZAR can predict the complex toxicological properties of compounds like mutagenicity, carcinogenicity, acute toxicity, maximum recommended daily dose, and blood–brain barrier penetration of chemical substances by applying data mining algorithms. This provides LAZAR with the advantage of providing toxicological predictions for endpoints that provide sufficient empirical information. The LAZAR approach prevents incomplete or incorrect background information from affecting the model predictions^30^. This web server is available from https://nano-lazar.in-silico.ch/predict. In the toxicological endpoints of chemical substances, mutagenicity and carcinogenicity are of great concern because of their serious effects on human health. Mutagenicity represents one of the most important end points of toxicity and also indicates stable changes in the DNA sequence of an organism, which may result in heritable changes in the characteristics of organisms^32^. The mechanism of action of mutagenic chemicals is mainly through DNA damage, which causes mutagenic effects such as chromosomal aberrations, frameshift mutations, and point mutations. Mutagens are not only involved in carcinogenesis and genotoxicity. They are also involved in the inception and pathogenesis of several chronic diseases including neurodegenerative disorders, diabetes, aging processes, arthritis, cardiovascular impairment, chronic inflammation, and hepatic disorders^33^. The LAZAR toxicity prediction accessible link is given in Table 1. LAZAR allows the prediction of several toxicological endpoints such as mutagenicity, rodent carcinogenicity blood–brain barrier penetration, and maximum recommended daily exposure of chemicals^34^.

**Table 1:** List of utilized web apps/servers.

S. no.	Web application	Server link
1	LAZAR	https://lazar.in-silico.ch/predict
2	PROTOX-II	https://tox-new.charite.de/protox_II/
3	CarcinoPred-EL	http://112.126.70.33/toxicity/CarcinoPred-EL/
4	BOILED-Egg	http://www.swissadme.ch/

### Honey bee toxicity prediction

VEGA extension allows to evaluate the properties of chemical substances. It allows to use all available VEGA QSAR models inside the analytical platform KNIME. The extension contains a node for the execution of VEGA models, taking as input a molecule set in SMILES format, and a further node to load and import structures from a SDF file. The model's results similarly to the text output from the VEGA stand-alone application, so that it is possible to use such results inside the workflow. VEGA offers a broad series of information, and the user should be aware of the elements offered by VEGA to assess a chemical substance. Considering all the evidence provided by VEGA the user may evaluate how credible a prediction is, and the level of uncertainty associated with the prediction. VEGA is a complex platform which basically includes a number of QSAR models, and an independent tool helping the user in the evaluation of the result, through an Applicability Domain Index. In this way VEGA combines QSAR and read across tools. The QSAR prediction models derive from CAESAR, T.E.S.T., SARpy, EPISuite, Toxtree, and other tools. New models are frequently added. Registered users are notified when new models are available. A completely independent algorithm is used to assess the reliability of the model prediction through what we call the Applicability Domain Index. This algorithm works on all the separate QSAR models. The algorithm shows similar compounds, assesses the QSAR results on similar compounds, and analyzes some relevant chemical features in the target compound and its related compounds. An automatic evaluation for read-across is done, and thus this tool can be used for read across, independently from the prediction obtained through the QSAR models. In the ideal situation both pieces of information, from the QSAR model and from the Applicability Domain Index, should be assessed. Hymenopteran insects, such as solitary bees, bumblebees and honey bees are important for their ecological services as pollinators of wild plants as well as economically important crops; they also provide honey and honey wax^35^. Toxicity prediction such as bee acute toxicity through the computational model KNN/IRFMN—v.1.0.0 was done via the downloadable JAVA based application VEGA version 1.1.4.

### Fish acute toxicity prediction

Acute toxicity assessments using fish are important for the risk assessments of environmental hazards of chemical compounds in aquatic systems by national legislation. Alternative methods like QSAR applications could substitute several ecotoxicity tests including experimental fish acute toxicity^36^. The acute fish toxicity model Sarpy/IRFMN v.1.0.2 accessed through the VEGA version 1.1.4. platform predicted fish acute toxicity in our study.

### Rodent oral toxicity prediction

The PROTOX-II webserver provides a prediction of rodent oral toxicity. For its application compound similarity analysis of median lethal doses (LD50) provided information about toxic fragments of given compounds^37^. ProTox-II is an online tool and was developed for toxicity prediction to design the development of the drug. This server can be accessed through a web link https://tox-new.charite.de/protox_II/. Animal trials are fundamental procedures for the determination of possible toxic effects of drug candidates and cosmetics. In silico prediction methods represent an alternative approach and aim to rationalize the preclinical drug development, thus enabling the reduction of the associated time, costs and animal experiments. In oral toxicity prediction, ProTox-II is working based on the analysis of the similarity of compounds with known median lethal doses (LD50) and incorporates the identification of toxic fragments, therefore, representing a novel approach in toxicity prediction. In addition, the web server includes an indication of possible toxicity targets which is based on an in-house collection of protein–ligand-based pharmacophore models (‘toxicophores’) for targets associated with adverse drug reactions. For the strong performance of ProTox-II (sensitivity, specificity and precision of 76, 95 and 75%, respectively) and superiority over other toxicity prediction tools, indicating their possible applicability for other compound classes^38^. Advancement in the field of computational research has made it possible for in silico methods to offer significant benefits to both regulatory needs and requirements for risk assessments, and the pharmaceutical industry to assess the safety profile of a chemical. Here, we present ProTox-II that incorporates molecular similarity, pharmacophores, fragment propensities and machine-learning models for the prediction of various toxicity endpoints; such as acute toxicity, hepatotoxicity, cytotoxicity, carcinogenicity, mutagenicity, immunotoxicity, adverse outcome pathways (Tox21) and toxicity targets. The predictive models are built on data from both in vitro assays (e.g. Tox21 assays, Ames bacterial mutation assays, hepG2 cytotoxicity assays, immunotoxicity assays) and in vivo data (e.g. carcinogenicity, hepatotoxicity). The webserver takes a two-dimensional chemical structure as an input and reports the possible toxicity profile of the chemical for 33 models with confidence scores, and an overall toxicity radar chart along with three most similar compounds with known acute toxicity^37^. ProTox-II incorporates the prediction of the various toxicities of the chemical which can be measured qualitatively as binary (active or inactive) in terms of endpoints such as acute toxicity, hepatotoxicity, carcinogenicity, immunotoxicity, mutagenicity, cytotoxicity, adverse outcome (toxicology in the twenty-first century) pathways, and toxicity targets^39^. It can further be measured quantitatively as 50% lethal dose (LD50) values. Toxicity classes (I–VI) can also be predicted based on toxic doses^40^. ProTox-II) https://tox-new.charite.de/protox_II (the web server is computational freely available in silico toxicity prediction tool consisting of 33 models to design the development of a drug). It incorporates the prediction of oral toxicities of chemicals which can be measured at different levels of toxicity mainly qualitatively in terms of endpoints as binary output (active or inactive) along with confidence scores such as acute toxicity, hepatotoxicity, carcinogenicity, immunotoxicity, mutagenicity, cytotoxicity, adverse outcome (Tox21) pathways, and toxicity (LD50) values in mg/kg body weight. Toxicity classes (Class I–VI) can also be predicted based on their toxic doses. Prediction accuracy, average similarity along with LD50, and toxicity class were generated instantly for the prediction of acute toxicity and toxicity targets. The phytochemicals and heterodimeric drug candidates were subjected to in silico pharmacodynamic studies to determine their potential cytotoxicity using the online web server ProTox-II (https://tox-new.charite.de/protox_II/, accessed on 28 November 2021)^37^. ProTox-II is a virtual lab for the predictive toxicity of small molecules, a process that can reduce the need for subsequent drug (in vivo) testing in animals. The ProTox-II server was used to determine predicted acute oral toxicity of the phytochemical compounds in rodents based upon 2D structure similarities with 38,000 compounds (provided by the software) and their associated LD50 values.

### Carcinogenicity prediction

Prediction of chemical carcinogenicity was attempted by using ensemble learning methods via CarcinoPred-EL. This web application accessible link is given in (Table 1). This application has three integrated ensemble models, namely Ensemble RF, SVM and XGBoost to provide carcinogenicity predictions. The ensemble learning method is commonly used to predict the carcinogenicity of chemicals^41^. Carcinogenicity refers to a highly toxic end point of certain chemicals, and has become an important issue in the drug development process. Three novel ensemble classification models, namely Ensemble SVM, Ensemble RF, and Ensemble XGBoost, were developed to predict carcinogenicity of chemicals using seven types of molecular fingerprints and three machine learning methods based on a dataset containing 1003 diverse compounds with rat carcinogenicity. More precisely in chemical datasets, the problem of imbalanced data is common. Most of the in silico models for the prediction of bioactivities as well as toxicity profiles had to rely on an imbalanced dataset^42^. In binary classification, the underrepresented class is generally referred to as minority class, and the over represented class is referred to as majority. It is often observed that in case of an asymmetric class distribution, regular classifiers like support vector machine (SVM), as well as neural network (NN) tend to ignore the minority class, and treat them as noise resulting in a class boundary that unduly benefits the majority class^43^. In CarcinoPred-EL among these three models (SVM,RF and XGBoost), Ensemble XGBoost is found to be the best, giving an average accuracy of 70.1 ± 2.9%, sensitivity of 67.0 ± 5.0%, and specificity of 73.1 ± 4.4% in five-fold cross-validation; an accuracy of 70.0%, sensitivity of 65.2%, and a specificity of 76.5% in external validation. In comparison with some recent methods, the ensemble models outperform some machine learning-based approaches and yield equal accuracy and higher specificity but lower sensitivity than rule-based expert systems. It is also found that the ensemble models could be further improved if more data were available. As an application, the ensemble models are employed to discover potential carcinogens in the DrugBank database. The results indicate that the proposed models are helpful in predicting the carcinogenicity of chemicals^41^. A web server called CarcinoPred-EL has been built for these models (http://112.126.70.33/toxicity/CarcinoPred-EL/index.html).

### Predicting gastrointestinal absorption and brain penetration of plasticizers

The *Brain OrIntestinaLEstimateD permeation* method (BOILED‐Egg) was utilized to investigate the gastrointestinal absorption and brain access property of plasticizers. This is a relatively precise prediction model that makes use of lipophilicity and polarity distributions of small molecules. This web application is commonly applied to predict the carcinogenicity of small molecules and lead compounds. We used it here for concomitant predictions of both brain and intestinal infiltration nature of plasticizers because of the accuracy, speed, conceptual simplicity and for the clear graphical output of BOILED-Egg. The Brain OrIntestinaLEstimateD (BOILED)-Egg permeation method is a predictive model that works with accuracy by computing the polarity and lipophilicity of small molecules and generates clear graphical outputs^48^. It is freely accessible from the Swiss ADME (http://www.swissadme.ch/). This tool is used to estimate various stages of a drug discovery process. Two pharmacokinetic characteristics play a crucial role, i.e., the prediction of gastrointestinal passive absorption and the permeability of the BBB^40^. According to BOILED-Egg plot analysis, compounds found in the yellow region were considered to have higher blood–brain barrier permeability, whereas compounds found in the white region of the plot were considered to have higher gastrointestinal absorption properties. The BOILED-Egg plot analysis was performed using the SwissADME web server^48^. The BOILED-Egg permeation method is a predictive model for the estimation of two pharmacokinetic behaviors, i.e., gastrointestinal passive absorption and the permeability of the blood–brain penetration barrier. It works with speed, accuracy, and conceptual ease by computing the polarity and lipophilicity of chemicals and generates clear graphical outputs^48^.

## Results and discussion

The LAZAR tool prediction for mutagenicity of tested plasticizers against the *Salmonella typhimurium* model indicates that the following bisphenols BPADE, BPAP, BPBP, BPF, BPM, and BPPH provide mutagenic effects. All other tested compounds did not show mutagenic properties (see S.Table 3). According to the published record, structural analogues of BPA, such as BPAF, BZP, BPS including BPF, do not provide mutagenic effects on the *Salmonella typhimurium* strains TA98 and TA100^44^, using the Ames assay for their evaluation of mutagenicity.

From the results of acute toxicity predictions on bees using the KNN/IRFMN v.1.0.0. model accessed through the VEGA version 1.1.4., DAP, DBP, DIHXP, and DPP were predicted as strong bee toxicants; DEP, DMP, BPADE, BPAF, BPC, BPF, and TBPA were moderately toxic and other test compounds were mildly toxic to bees (S.Table 4). For DBP there were no chronic toxicity data available from honey bees. However, during the present computational study, DBP was a strong bee toxicant predicted by the VEGA software. Based on predicted results of fish acute toxicity, Sarpy/IRFMN-1.0.2 classifies the toxic compounds in toxic classes 1, 2, 3 etc. For example, if an LC50 value was less than 1 mg/L, such a compound was classified as a class 1 toxicant. BBP, BCP, BDP, DEHP, DBP, DCP, DIDP, DIHPP, DIHXP, DINP, DIOP, DITP, DIUP, DMP, DNHP, DNPP, DPHP, DTDP, DUP, ODP, DEHP, and DNOP were class 1 toxicants in the fish acute toxicity model. BPM, BPP, BPPH, and TBPA were class 2 toxicants to fish. Other tested compounds were classified as class 3 toxicants according to the Sarpy/IRFMN-1.0.2 fish acute toxicity model (S.Table 5). Fish acute toxicity of BBP in shiner perch (*Cymatogaster aggregate*) may affect its central adrenergic nervous system^45^.

Carp (*Cyprinus carpio*) exposed to DBP and DEHP separately and combined exposure of DBP and DEHP are affected and these two plasticizers provide an ecological risk^36^. The PROTOX-II web server predicted BCP, DEP, DIBP, DIHPP, DIHXP, DNHP, DNPP, DPP, BPADE, BPG, and TBPA as least toxic from all compounds tested in the present study. BBP, DBP, BPAF, BPM, and BPPH were predicted as class 5 toxicants and other tested compounds were predicted as class 4 toxicants during rodent oral toxicity testing (S.Table 6). Carcinogenicity of plasticizers was predicted by using ensemble learning methods of CarcinoPred-EL^46^. It predicted BBP, BCP, BDP, DBP, DEHP, DCP, DIDP, DIHPP, DIHXP, DINP, DIOP, DITP, DIUP, DNHP, DNPP, DPHP, DTDP, DUP, ODP, BPADE, DEHP, and DNOP as carcinogens in at least one of the CarcinoPred-EL ensemble models. This tool was developed based on animal experimental data. All other tested compounds were non-carcinogenic.

Whereas carcinogenicity of DBP was not studied in humans as yet, BBP was predicted as carcinogenic in humans^47^ (S.Table 7). However, based on Toxnet data, DBP is a liver carcinogen for rodents. According to the CarcinoPred-EL tool, DBP was predicted as a carcinogen. DEHP may induce cancer in humans and rodents through multiple molecular signaling pathways^48^. Carcinogenic effects were not conclusively determined for DNO. But, CarcinoPred-EL predicted DNOP as a carcinogen with the RF and XGBoost model which were developed using animal experimental data. The carcinogen predictive tool was not developed from human experimental data. Data were derived from cancer inducing experiments with animal models. Surely, are tumors not only species- but even organ- and tissue-specific. Their modes of development or prevention, and even their diagnosis through appropriate biomarkers is expected to be very different from the distinctions above.

**Figure 2:**
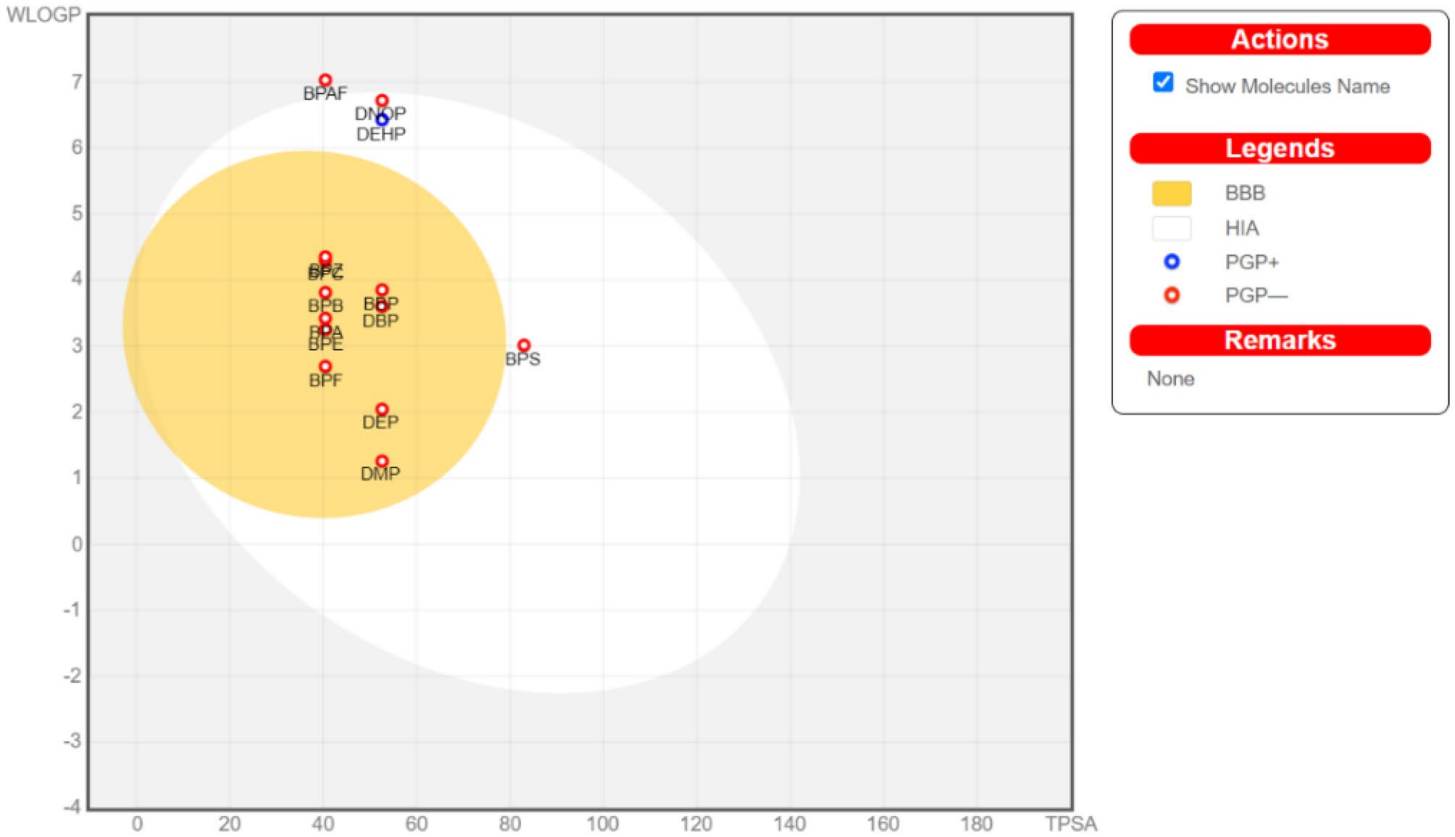
Prediction of gastrointestinal absorption and brain penetration of plasticizers, BOILED-EGG accessed through SwissADME (http://www.swissadme.ch/).

The plasticizer DEHP alone can provide a P-gp substrate (PGP+) (Points located within BOILED-Egg's yolk PGP+ predicted to be effluating from the Central Nervous System (CNS) by the P-glycoprotein) (Fig. 2); for more details see also S.Fig. 2. But all other test compounds were not PGP−. DEHP can get absorbed to the blood stream in the human gastrointestinal tract. Also, DEHP, BPAF, DNOP, and BPS are predicted as HIA (points located in BOILED-Egg's yolk, HIA predicts as to be passively absorbed by the gastrointestinal tract.). These plasticizers are not entering the human blood–brain barrier (BBB) with results provided in Annexure 1 and 2.

## Conclusions

Computational tools provide a fast and cost-effective screening of toxicological characteristics of plasticizers. Based on the LAZAR mutagenicity assessment were bisphenol F alone predicted as mutagenic. The BBP and DBP in RF; DEHP in RF and XGBoost; DNOP in RF and XGBoost models were predicted as carcinogenic in the CarcinoPred-EL web application. From the bee predictive model (KNN/IRFMN) BPF, di-n-propyl phthalate, diallyl phthalate, dibutyl phthalate, and diisohexyl phthalate were predicted as strong bee toxicants. Acute toxicity for fish using the model Sarpy/IRFMN predicted 19 plasticizers as strong toxicants with LC50 values of less than 1 mg/L. This study provides predicted data of plasticizers on gastrointestinal absorption and other toxicological endpoints. Web-based in silico tools and VEGA were used in this study for the prediction of toxicological properties of plasticizers in order to reduce and replace toxicological in vivo and in vitro experiments and to deliver early alerts for environmental and public health risks. The web tools used here turned out to be fast and reliable and were open access.

## Supplementary Information

Supplementary Information 1.

Supplementary Information 2.

Supplementary Figures.

Supplementary Tables.

## Data Availability

Most data generated or analyzed during this study are included in this published article [and its ANNEX information files]. The complete datasets used and/or analyzed during the current study is available from the corresponding author upon reasonable request.
