# Supplementary material for: Evaluating different web applications to assess the toxicity of plasticizers
Source: Sci Rep. 2022 Nov 16;12:19684. doi: 10.1038/s41598-022-18327-0 (PMC9668977; doi:10.1038/s41598-022-18327-0)
Supplement: Supplementary file 2 — Supplementary Information 2. [file 41598_2022_18327_MOESM2_ESM.docx]

**Annexure**

1. **Technical details of the utilized *in silico* tools**

Increase in the number of new chemicals synthesized in the past decades has resulted in constant growth and the development and application of computational models for the prediction of activity as well as safety profiles of the chemicals (Banerjee et al., 2018; Mitchell, 2014; Banerjee et al., 2016; Hong et al., 2017). *In silico* models based on quantitative structure activity relationship modeling to molecular similarity-based methods and machine learning models have been greatly successful in the field of computational drug design (Huang et al., 2016, Banerjee et al., 2016).

1. **Selection of chemicals for computational analysis**

Phthalic acid esters (PAEs) are used as plasticizer or modifiers in artificially-manufactured products. Though the rapid biotransformation of phthalates in microbes and plants have been well documented, it is less studied yet in terrestrial animals, e.g. earthworm (Fan et al., 2022). The probability of nonoccupational exposure to phthalates is high given their use in a vast range of consumables, including personal care products (e.g., perfumes, lotions, cosmetics), paints, industrial plastics, and certain medical devices and pharmaceuticals. Phthalates are of high interest because of their potential for human exposure and because animal toxicity studies suggest that some phthalates affect male reproductive development apparently via inhibition of androgen biosynthesis. In humans, phthalates are rapidly metabolized to their monoesters, which can be further transformed to oxidative products, conjugated, and eliminated. Phthalate metabolites have been used as biomarkers of exposure. Using urinary phthalate metabolite concentrations allowing the accurate assessment of human exposure because these concentrations represent an integrative measure of exposure to phthalates from multiple sources and routes. However, the health significance of this exposure is unknown (Calafat and McKee, 2006).

***SMILES preparation***

Molecular structures were represented by the strings of special characters using SMILES as kernel strings. Since SMILES-based similarity functions are computationally more efficient, we used a SMILES-based toxicity prediction (Öztürk et al., 2016; Frenzel et al., 2017).

***Benzyl butyl phthalate***

Butyl benzyl phthalate (BBP) has been extensively used worldwide as a plasticizer in the polyvinyl chloride (PVC) industry and the manufacturing of many other products, and its presence in the aquatic environment is expected for decades (Herrero et al., 2015).

***Dibutyl phthalate***

Dibutyl phthalate (DBP) is a most widely used plasticizer for agricultural mulching films and one of the most common organic pollutants in black soil.The DBP is directly or indirectly affect ammonia oxidizing bacteria (AOB) and nitrite oxidizing bacteria (NOB) through three ways (Tao et al., 2022). DBP is one of the most commonly used and toxic phthalate esters and has a variety of harmful effects on aquatic animals. However, there is still a lack of knowledge on the accumulation, detoxification, and toxicity of DBP in aquatic animals. In swimming crab *Portunustrituberculatus*, DBP exposure induced the different responses of three cytochrome P450 members and antioxidant enzyme genes, enhanced gene transcript and protein levels of glutathione-S-transferase and two heat stress proteins and malondialdehyde accumulation, decreased glutathione level, and inhibited antioxidant enzyme activities. Furthermore, no significant effect of DBP was observed on crab survival, size, and weight but molting retardation was observed. Therefore, DBP induced strong detoxification and antioxidative defense mechanisms to overcome detrimental effects of DBP on swimming crab juveniles despite a molting retardation as a trade-off in fitness costs (He et al., 2022).

***Diethyl phthalate***

Diethyl phthalate (DEP; CAS No. 84-66-2) has several industrial applications, as a solvent and vehicle for fragrance and cosmetic ingredients and subsequent skin contact (Api, 2001).

***di-(2-ethylhexyl)phthalate***

Numerous epidemiological findings have shown that di-(2-ethylhexyl)-phthalate (DEHP), one of the industrial plasticizers with endocrine-disrupting properties, positively contributes to high incidence of obesity. However, potential pathogenesis of dietary DEHP exposure-induced obesity remains largely unknown (Su et al., 2022). Plasticizers such as di(2-ethylhexyl) phthalate (DEHP)manufactured chemicalsare produced in high quantities. DEHP is frequently detected in the aquatic environment causing toxic effects on organisms (Dao et al., 2022). DEHP is an endocrine-disrupting chemical that induces numerous health problems when present in the human body in trace amounts (Lee et al., 2022). Di (2-ethylhexyl) phthalate (DEHP), classified as a reproductive toxicant, is a ubiquitous pollutant in foodstuffs, dust, and commercial products (Yoon et al., 2022). DEHP is suggested to potentially induce the epigenetic alterations that might increase the risk of breast cancer, given that the underlying mechanisms should be fully elucidated. Leaching of the plastic constituents leading to their chronic exposure to humans is a major concern forenvironmental and occupational health. The studies of Ghosh et al. (2022) have demonstrated that environmental chemicals like di (2-Ethylhexyl)-phthalate (DEHP) could pose a risk towards epigenetic mechanisms (Ghosh et al., 2022).Zheng et al. (2022) demonstrate that DEHP may cause biochemical and genetic toxicity to *Folsomia candida*, which illustrated the potential risks of DEHP to soil environmentsthat might affect soil ecosystem processes (Zheng etal., 2022). Further studies are necessary to elucidate the toxic mechanisms of DEHP on other non-target organisms in soil (Zheng etal., 2022).

***dimethyl phthalate***

Dimethyl phthalate (DMP) is a endocrine disruptor and one of the phthalate esters (PAEs). This ubiquitous pollutant and it impacts on living organisms have aroused great concern. In 2015,Wang *et al*. showed that the operational taxonomic unit (OTUs) richness and bacterial diversity were reduced in DMP contaminated soil. And the relative percentages of some genera associated with nitrogen metabolism were increased by DMP contamination, while the relative percentages of some other genera that were extremely beneficial to soil health were decreased by DMP contamination. Furthermore, the relative percentages of some genera that possessed the capability to degrade DMP were increased by the DMP treatment at low concentrations (5, 10, and 20 mg/kg), but were decreased by high concentration DMP treatments (40 mg/kg). Clearly, DMP contamination changed the bacterial community structure and disturbed the metabolic activity and functional diversity of the microbes in black soils. Wang *et al*., 2015 results suggest that DMP pollution can alter the metabolism and biodiversity of black soil microorganisms, thereby directly impact fertility and ecosystem functions (Wang et al., 2015).

***dioctyl phthalate***

Phthalates are endocrine disrupting chemicals that may influence the weight status since some studies have considered weight gain during pregnancy and subsequent long-term weight changes in women. Prenatal phthalate exposure was inversely associated with gestational weight gain and positively associated with long-term changes in maternal weight. Further investigations are required to understand how phthalates may influence body composition and whether they contribute to the development of obesity and cardiometabolic diseases in women (Deierlein et al., 2022).

***bisphenol A***

As an endocrine disruptor, bisphenol A (BPA) is a severe threat to human health (Li et al., 2022). This endocrine disrupting chemicals used in consumer products, building materials, and food processing and packaging materials. They are associated with adverse health outcomes, especially when exposure occurs during heightened windows of susceptibility (Smith *et al*., 2022). BPA can disrupt the production, function and activity of endogenous hormones causing irregularity in the hypothalamus-pituitary-gonadal glands and also the pituitary-adrenal function. BPA has immuno-suppression activity and can downregulate T cells and antioxidant genes (Tarafdar et al., 2022).

***bisphenol AF***

Bisphenol AF (BPAF) is an emerging contaminant prevalent in the environment as one of main substitutes of bisphenol A (BPA). It was found that BPAF exhibited estrogenic effects in zebrafish larvae in the study of Chen et al. (2022), while little is known about its effects on the thyroid and liver. A 7 d zebrafish embryotoxicity test was conducted to study the potential thyroid disruption and hepatotoxicity of BPAF (Chen et al., 2022).Liu et al. (2021) reported that bisphenol AF (BPAF) works as an agonist for estrogen receptor (ER) ERα but as an antagonist for ERβ. Similar results were observed for bisphenol E analogs (BPE-X) such as BPE-F, BPE-Cl, and BPE-Br, each consisting of a series of a tri-halogenated methyl group CX_3_ in the central alkyl moiety(Liu et al., 2021).

***bisphenol B***

Bisphenol B (BPB) has been used as a substitute for bisphenol A (BPA) in plastic materials. Whether BPB disrupts the male reproductive system remains unknown.The study of Li et al. (2021) showedthat BPB significantly increased Leydig cell number at 100 and 200 mg/kg, while down-regulating the expression of Leydig cell genes (Cyp11a1 and Hsd3b1) at ≥100 mg/kg and up-regulating the expression of Sertoli cell genes (Pdgfra, Fshr, Sox9) and cell cycle regulators (Pcna, Ccnb1, Cdk2, and Cdk4) at 10–200 mg/kg (Li et al., 2021). BPB markedly increased the phosphorylation of AKT1, AKT2, and ERK1/2 at 200 mg/kg. BPB increased the proliferation of rat immature Leydig cells via promoting the S/M2 phase shift at 100 and 1000 nM after 24-h culture in vitro(Li et al., 2021). In conclusion, BPB disrupts Leydig cell maturation in late puberty by increasing the Leydig cell number while inhibiting its maturation (Li et al., 2021). BPB is recognized as a potential endocrine disrupting chemical owing to its estrogenic and anti-androgenic nature (Ikhlas et al., 2021). Therefore, it was pertinent to study the effect of BPB exposure during the adolescence age (5–6 weeks old) in male mice (Ikhlas et al., 2021). Weekly intraperitoneal injections of 5, 10 and 15% LD_50_ of BPB were given for 2 weeks to acute exposure groups and for 4 weeks to sub-acute exposure groups (Ikhlas et al., 2021). BPB exposure induces change in enzymatic and non-enzymatic oxidative stress markers in sperm samples. DNA damage was also observed in sperm cells on acute and sub-acute exposures. Furthermore, BPB exposure led to a marked decline in sperm count and compromised sperm morphology (Ikhlas et al., 2021). Computer assisted sperm analysis (CASA) revealed a significant decrease in sperm quality and progressive motility (Ikhlas et al., 2021). Thus, both the acute and sub-acute exposures of adolescent male mice to BPB adversely affect the sperms quality, function and morphology (Ikhlas et al., 2021).

***bisphenol E***

Bisphenol E (bis (4-hydroxyphenyl) ethane, BPE), as a typical endocrine disrupting chemical, is commonly detected in source water and drinking water, which poses potential risks to human health and the ecological environment (Tian et al., 2021). Bisphenol E (BPE) have recently replaced bisphenol A as monomers for producing polycarbonates (Coha et al., 2021). However, BPE can pose hazards as they are known to be endocrine disruptors (Coha et al., 2021). Despite the huge increase in their use, there is a lack of data regarding the toxicity and effects of BPE (Coha et al., 2021). Tian et al., 2021 collected samples from river, tap water, synthetic wastewater, and secondary effluent were tested to explore the feasibility of Fe(VI) oxidation for treating BPE in water. It was found that 99% of BPE was degraded within 300 s in these waters except for synthetic wastewater (Tian et al., 2021). The toxicity of BPE and its intermediates was evaluated by ECOSAR program, and the results showed that Fe(VI) oxidation decreased the toxicity of reaction solutions (Tian et al., 2021). These findings demonstrated that the Fe(VI) oxidation process was an efficient and green method for the treatment of BPE, and the new insights into the double-oxygen transfer mechanism aid to understand the reaction mechanisms of organic pollutants oxidized by Fe(VI) (Tian et al., 2021).

***bisphenol C***

Chemicals that disrupt androgen receptor (AR) function *in utero* induce a cascade of adverse effects in male rats including reduced anogenital distance, retained nipples, and reproductive tract malformations. From the experiment of Gray et al. (2019) *In vitro*, BPC was as potent an AR antagonist as hydroxyflutamide. Furthermore, BPC inhibited fetal testis testosterone production and testis gene expression *ex vivo*. However, when BPC was administered at 100 and 200 mg/kg/d *in utero*, the reproductive tract of the male offspring was minimally affected. None of the males displayed reproductive malformations. For comparison, *in utero* administration of flutamide has been shown to induce malformations in 100% of males at 6 mg/kg/d. *In vitro*, PFQ was several orders of magnitude less potent than BPC, vinclozolin, or procymidone. However, *in utero* administration of 12.5, 25, 50, and 100 mg PFQ/kg/d on GD 14–18 induced antiandrogenic effects at all dosage levels and 91% of the males displayed reproductive malformation in the high dose group. Overall, BPC was ∼380-fold more potent than PFQ *in vitro*, whereas PFQ was far more potent than BPC *in utero*. Incorporating toxicokinetic and toxicodynamic data into *in vitro* to *in vivo* extrapolations would reduce the discordance between the *in vitro* and *in utero* effects of PFQ and BPC and combining *in vitro* results with a short-term Hershberger assay would reduce the uncertainty in predicting the *in utero* effects of antiandrogenic chemicals (Gray et al., 2019).

***bisphenol F***

Bisphenol F (BPF), as an important bisphenol A substitute, is being increasingly used for industrial production. Mu et al. (2022), performed large scale fecundity test for zebrafish that are continuous exposed to environmental levels of BPF (0.5, 5 and 50 μg/L) from embryonic stage, and identified suppressed spawning capacity of females and reduced fertility rate of males in adulthood. Although pathological change is only observed in female gonads, the transcriptional change in the hypothalamic-pituitary-gonad axis genes occurred in the gonads of both female and male fish at 150 days post-exposure. F1 generation embryos showed abnormal developmental outcomes including decreased heart rate, reduced body length, and inhibition of spontaneous movement after parental exposure to BPF. RNA-sequencing showed that the genes involved in skeletal/cardiac muscle development were significantly altered in F1 embryos spawned by BPF-treated zebrafish. Advanced pathway analysis showed that cancer and tumour formation were the most enriched pathways in the offspring of 0.5 and 5.0 μg/L groups; organismal development and cardiovascular system development were mainly affected after parental exposure to 50 μg/L of BPF; these changes were mediated by several involved regulators such as GATA4, MYF6, and MEF2C. These findings confirmed that long-term exposure to BPF at environmentally relevant concentrations would result in reproductive toxicity among zebrafish indicating the urgent demand for the control of BPA substitutes (Mu et al., 2022).

***bisphenol S***

Bisphenol S (BPS), an increasingly used alternative to bisphenol A, has been linked to testosterone deficiency and male reproductive dysfunction in laboratory animals. Zhang et al., 2022 observed, after treatment with BPS (100, 200 and 400 μM) for 48 h in vitro, TM3 mouse Leydig cells exhibited a dose-dependent decrease in their viability. Furthermore, BPS challenge triggered oxidative stress manifested by compromised activities of superoxide dismutase and catalase with exaggerated formation of reactive oxygen species (Zhang et al., 2022). Especially, BPS exposure resulted in augmented mitochondrial permeability transition pore opening, dissipated mitochondrial membrane potential and reduced ATP generation, along with an altered energy metabolism (Zhang et al., 2022). Moreover, BPS stimulation enhanced BAX expression and caspase-3 activity and inhibited BCL-2 expression. In addition, BPS-treated TM3 cells showed an accumulation of autophagic vacuoles, together with increased Beclin1 and P62 expression and elevated LC3B-II/LC3B-I ratio. These results demonstrated that in vitro exposure to BPS exerted cytotoxicity to TM3 Leydig cells through inducing oxidative stress, mitochondrial impairment, autophagic disturbance and apoptosis (Zhang et al., 2022).

***bisphenol Z***

The chemical nomenclature of BPZ is 1,1-bis (4-hydroxyphenyl) cyclohexane. BP compounds including BPZ have been found in different environmental and human samples. BPZ may be used in the synthesis of an anesthetic compound. Another area of use of BPZ is to cure highly heat resistant plastic materials and in electrical insulation (Kovačič et al., 2019). Schmidt et al. 2013 showed that BPZ was biotransformed in highest level of mobility (HLM) in a manner similar to the case of BPAF (Schmidt et al., 2013). According to the research based on the peak areas, hydroxylated BPZ was the main *in vitro* metabolite in HLM in the presence of nicotinamide adenine dinucleotide phosphate and GSH.

**References**

Riyad, P., Purohit, A., Karishma, S. and Ram, H., 2022. Atherosclerotic plaque regression and HMG-CoA reductase inhibition potential of curcumin: An integrative omics and *in-vivo* study. *Journal of Applied Biology & Biotechnology Vol*, *10*(01), pp.129-135.

Zhang, W., Huang, T., Sun, Z., Kuang, H., Yuan, Y., Zou, W., Liu, F., Zhang, F., Yang, B., Wu, L. and Zhang, D., 2022. Bisphenol S exposure induces cytotoxicity in mouse Leydig cells. *Food and Chemical Toxicology*, p.112805.

Kovačič, A., Gys, C., Kosjek, T., Covaci, A. and Heath, E., 2019. Photochemical degradation of BPF, BPS and BPZ in aqueous solution: Identification of transformation products and degradation kinetics. *Science of The Total Environment*, *664*, pp. 595-604.

Schmidt, J., Kotnik, P., Trontelj, J., Knez, Ž. and Mašič, L.P., 2013. Bioactivation of bisphenol A and its analogs (BPF, BPAF, BPZ and DMBPA) in human liver microsomes. *Toxicology in Vitro*, *27*(4), pp.1267-1276.

Chen, P., Wang, R., Chen, G., An, B., Liu, M., Wang, Q. and Tao, Y., 2022. Thyroid endocrine disruption and hepatotoxicity induced by bisphenol AF: Integrated zebrafish embryotoxicity test and deep learning. *Science of The Total Environment*, p.153639.

Coha, M., Dal Bello, F., Fabbri, D., Calza, P. and Medana, C., 2021. Structural elucidation of bisphenol E and bisphenol S photoinduced by‐products by high‐resolution electrospray ionisation mass spectrometry and tandem mass spectrometry. *Rapid Communications in Mass Spectrometry*, *35*(7), p.e9039.

Tian, B., Wu, N., Pan, X., Yan, C., Sharma, V.K. and Qu, R., 2021. Ferrate (VI) oxidation of bisphenol E: Kinetics, removal performance, and dihydroxylation mechanism. *Water Research*, p.118025.

Wang, Z.G., Hu, Y.L., Xu, W.H., Liu, S., Hu, Y. and Zhang, Y., 2015. Impacts of dimethyl phthalate on the bacterial community and functions in black soils. *Frontiers in Microbiology*, *6*, p.405.

Mu, X., Qi, S., Liu, J., Wang, H., Yuan, L., Qian, L., Li, T., Huang, Y., Wang, C., Guo, Y. and Li, Y., 2022. Environmental level of bisphenol F induced reproductive toxicity toward zebrafish. *Science of The Total Environment*, *806*, p.149992.

Gray Jr, L.E., Furr, J.R., Conley, J.M., Lambright, C.S., Evans, N., Cardon, M.C., Wilson, V.S., Foster, P.M. and Hartig, P.C., 2019. A conflicted tale of two novel AR antagonists *in vitro* and *in vivo*: Pyrifluquinazon *versus* bisphenol C. *Toxicological Sciences*, *168*(2), pp.632-643.

Liu, X., Suyama, K., Nose, T., Shimohigashi, M. and Shimohigashi, Y., 2021. Bisphenol-C is the strongest bifunctional ERα-agonist and ERβ-antagonist due to magnified halogen bonding. *Plos one*, *16*(2), p.e0246583.

Li, Y., Yan, H., Yu, Y., Zou, C., Tian, L., Xin, X., Zhang, S., Li, Z., Ma, F. and Ge, R.S., 2021. Bisphenol B stimulates Leydig cell proliferation but inhibits maturation in late pubertal rats. *Food and Chemical Toxicology*, *153*, p.112248.

Ikhlas, S. and Ahmad, M., 2020. Acute and sub-acute bisphenol-B exposures adversely affect sperm count and quality in adolescent male mice. *Chemosphere*, *242*, p.125286.

Tao, Y., Feng, C., Xu, J., Shen, L., Qu, J., Ju, H., Yan, L., Chen, W. and Zhang, Y., 2022. Di (2-ethylhexyl) phthalate and dibutyl phthalate have a negative competitive effect on the nitrification of black soil. *Chemosphere*, p.133554.

He, Y., Lin, W., Shi, C., Li, R., Mu, C., Wang, C. and Ye, Y., 2022. Accumulation, detoxification, and toxicity of dibutyl phthalate in the swimming crab. *Chemosphere*, *289*, p.133183.

Api, A.M., 2001. Toxicological profile of diethyl phthalate: a vehicle for fragrance and cosmetic ingredients. *Food and Chemical Toxicology*, *39*(2), pp.97-108.

Calafat, A.M. and McKee, R.H., 2006. Integrating biomonitoring exposure data into the risk assessment process: phthalates [diethyl phthalate and di (2-ethylhexyl) phthalate] as a case study. *Environmental Health Perspectives*, *114*(11), pp.1783-1789.

Banerjee, P., Dehnbostel, F.O. and Preissner, R., 2018. Prediction is a balancing act: importance of sampling methods to balance sensitivity and specificity of predictive models based on imbalanced chemical data sets. *Frontiers in Chemistry*, *6*, p.362.

Mitchell, J.B., 2014. Machine learning methods in chemoinformatics. *Wiley Interdisciplinary Reviews: Computational Molecular Science*, *4*(5), pp.468-481.

Herrero, Ó., Planelló, R. and Morcillo, G., 2015. The plasticizer benzyl butyl phthalate (BBP) alters the ecdysone hormone pathway, the cellular response to stress, the energy metabolism, and several detoxication mechanisms in *Chironomus riparius* larvae. *Chemosphere*, *128*, pp.266-277.

Daina, A. and Zoete, V., 2016. A boiled‐egg to predict gastrointestinal absorption and brain penetration of small molecules. *ChemMedChem*, *11*(11), p.1117.

Tian, S., Wang, J., Li, Y., Li, D., Xu, L. and Hou, T., 2015. The application of in silico drug-likeness predictions in pharmaceutical research. *Advanced Drug Delivery Reviews*, *86*, pp.2-10.

Drwal, M.N., Banerjee, P., Dunkel, M., Wettig, M.R. and Preissner, R., 2014. ProTox: a web server for the *in silico* prediction of rodent oral toxicity. *Nucleic Acids Research*, *42*(W1), pp.W53-W58.

Li, Z., Luo, C., Tan, F., Wu, D., Zhai, X., Wang, S., Cheng, X., Zhang, F., Li, M. and Ma, Q., 2022. UV-light irradiation combined with nitrate for degradation of bisphenol A: kinetics, transformation pathways, and acute toxicity assessment. *Environmental Science: Water Research & Technology*.

Tarafdar, A., Sirohi, R., Balakumaran, P.A., Reshmy, R., Madhavan, A., Sindhu, R., Binod, P., Kumar, Y., Kumar, D. and Sim, S.J., 2022. The hazardous threat of Bisphenol A: Toxicity, detection and remediation. *Journal of Hazardous Materials*, *423*, p.127097.

Smith, A.R., Kogut, K.R., Parra, K., Bradman, A., Holland, N. and Harley, K.G., 2022. Dietary intake and household exposures as predictors of urinary concentrations of high molecular weight phthalates and bisphenol A in a cohort of adolescents. *Journal of Exposure Science & Environmental Epidemiology*, *32*(1), pp.37-47.

Banerjee, P., Eckert, A.O., Schrey, A.K. and Preissner, R., 2018. ProTox-II: a webserver for the prediction of toxicity of chemicals. *Nucleic Acids Research*, *46*(W1), pp.W257-W263.

Mitchell, J.B., 2014. Machine learning methods in chemoinformatics. *Wiley Interdisciplinary Reviews: Computational Molecular Science*, *4*(5), pp.468-481.

Banerjee, P., Siramshetty, V.B., Drwal, M.N. and Preissner, R., 2016. Computational methods for prediction of in vitro effects of new chemical structures. *Journal of Cheminformatics*, *8*(1), pp.1-11.

Hong, H., Thakkar, S., Chen, M. and Tong, W., 2017. Development of decision forest models for prediction of drug-induced liver injury in humans using a large set of FDA-approved drugs. *Scientific Reports*, *7*(1), pp.1-15.

Huang, R., Xia, M., Sakamuru, S., Zhao, J., Shahane, S.A., Attene-Ramos, M., Zhao, T., Austin, C.P. and Simeonov, A., 2016. Modelling the Tox21 10 K chemical profiles for *in vivo* toxicity prediction and mechanism characterization. Nat Commun 7: 10425.

Banerjee, P., Siramshetty, V.B., Drwal, M.N. and Preissner, R., 2016. Computational methods for prediction of *in vitro* effects of new chemical structures. *Journal of Cheminformatics*, *8*(1), pp.1-11.

López, V., Fernández, A., García, S., Palade, V. and Herrera, F., 2013. An insight into classification with imbalanced data: Empirical results and current trends on using data intrinsic characteristics. *Information Sciences*, *250*, pp.113-141.

Li, D.C., Liu, C.W. and Hu, S.C., 2010. A learning method for the class imbalance problem with medical data sets. *Computers in Biology and Medicine*, *40*(5), pp.509-518.

Nanni, L., Fantozzi, C. and Lazzarini, N., 2015. Coupling different methods for overcoming the class imbalance problem. *Neurocomputing*, *158*, pp.48-61.

Maltarollo, V.G., Gertrudes, J.C., Oliveira, P.R. and Honorio, K.M., 2015. Applying machine learning techniques for ADME-Tox prediction: a review. *Expert Opinion on Drug Metabolism & Toxicology*, *11*(2), pp.259-271.

Drwal, M.N., Siramshetty, V.B., Banerjee, P., Goede, A., Preissner, R. and Dunkel, M., 2015. Molecular similarity-based predictions of the Tox21 screening outcome. *Frontiers in Environmental Science*, *3*, p.54.

Stefaniak, F., 2015. Prediction of compounds activity in nuclear receptor signaling and stress pathway assays using machine learning algorithms and low-dimensional molecular descriptors. *Frontiers in Environmental Science*, *3*, p.77.

Capuzzi, S.J., Politi, R., Isayev, O., Farag, S. and Tropsha, A., 2016. QSAR modeling of Tox21 challenge stress response and nuclear receptor signaling toxicity assays. *Frontiers in Environmental Science*, *4*, p.3.

Fan, X., Gu, C., Cai, J., Zhong, M., Bian, Y. and Jiang, X., 2022. Mechanistic insights into primary biotransformation of diethyl phthalate in earthworm and significant SOD inhibitory effect of esterolytic products. *Chemosphere*, *288*, p.132491.

Mayr, A., Klambauer, G., Unterthiner, T. and Hochreiter, S., 2016. DeepTox: toxicity prediction using deep learning. *Frontiers in Environmental Science*, *3*, p.80.

Banerjee, P. and Preissner, R., 2018. BitterSweetForest: a random forest based binary classifier to predict bitterness and sweetness of chemical compounds. *Frontiers in Chemistry*, *6*, p.93.

Dubey, R., Zhou, J., Wang, Y., Thompson, P.M., Ye, J. and Alzheimer's Disease Neuroimaging Initiative, 2014. Analysis of sampling techniques for imbalanced data: An n= 648 ADNI study. *NeuroImage*, *87*, pp.220-241.

Beyan, C. and Fisher, R., 2015. Classifying imbalanced data sets using similarity based hierarchical decomposition. *Pattern Recognition*, *48*(5), pp.1653-1672.

Pérez, G.M., Salomón, L.A., Montero-Cabrera, L.A., de la Vega, J.M.G. and Mascini, M., 2016. Integrating sampling techniques and inverse virtual screening: toward the discovery of artificial peptide-based receptors for ligands. *Molecular Diversity*, *20*(2), pp.421-438.

López, V., Fernández, A., García, S., Palade, V. and Herrera, F., 2013. An insight into classification with imbalanced data: Empirical results and current trends on using data intrinsic characteristics. *Information Sciences*, *250*, pp.113-141.

Huang, R., Xia, M., Sakamuru, S., Zhao, J., Shahane, S.A., Attene-Ramos, M., Zhao, T., Austin, C.P. and Simeonov, A., 2016. Modelling the Tox21 10 K chemical profiles for in vivo toxicity prediction and mechanism characterization. *Nat Commun* 7: 10425.

Chen, M., Suzuki, A., Thakkar, S., Yu, K., Hu, C. and Tong, W., 2016. DILIrank: the largest reference drug list ranked by the risk for developing drug-induced liver injury in humans. *Drug Discovery Today*, *21*(4), pp.648-653.

Thakkar, S., Chen, M., Fang, H., Liu, Z., Roberts, R. and Tong, W., 2018. The Liver Toxicity Knowledge Base (LKTB) and drug-induced liver injury (DILI) classification for assessment of human liver injury. *Expert Review of Gastroenterology & Hepatology*, *12*(1), pp.31-38.

Zhang, L., Ai, H., Chen, W., Yin, Z., Hu, H., Zhu, J., Zhao, J., Zhao, Q. and Liu, H., 2017. CarcinoPred-EL: novel models for predicting the carcinogenicity of chemicals using molecular fingerprints and ensemble learning methods. *Scientific Reports*, *7*(1), pp.1-14.

Su, H., Yuan, P., Lei, H., Zhang, L., Deng, D., Zhang, L. and Chen, X., 2022. Long-term chronic exposure to di-(2-ethylhexyl)-phthalate induces obesity via disruption of host lipid metabolism and gut microbiota in mice. *Chemosphere*, *287*, p.132414.

Zheng, Y., Zhou, K., Tang, J., Liu, C. and Bai, J., 2022. Impacts of di-(2-ethylhexyl) phthalate on *Folsomia candida* (Collembola) assessed with a multi-biomarker approach. *Ecotoxicology and Environmental Safety*, *232*, p.113251.

Dao, T.S., Nguyen, V.T., Baduel, C., Bui, M.H., Tran, V.T., Pham, T.L., Bui, B.T. and Dinh, K.V., 2022. Toxicity of di-2-ethylhexyl phthalate and tris (2-butoxyethyl) phosphate to a tropical micro-crustacean (Ceriodaphniacornuta) is higher in Mekong River water than in standard laboratory medium. *Environmental Science and Pollution Research*, pp.1-13.

Ghosh, K., Chatterjee, B., Nalla, K.K., Behera, B., Mukherjee, A. and Kanade, S.R., 2022. Di-(2-ethylhexyl) phthalate triggers DNA methyltransferase 1 expression resulting in elevated CpG-methylation and enrichment of MECP2 in the p21 promoter in vitro. *Chemosphere*, p.133569.

Lee, K., Gurudatt, N.G., Heo, W., Hyun, K.A. and Jung, H.I., 2022. Ultrasensitive detection and risk assessment of di (2-ethylhexyl) phthalate migrated from daily-use plastic products using a nanostructured electrochemical aptasensor. *Sensors and Actuators B: Chemical*, p.131381.

Yoon, H., Kim, T.H., Lee, B.C., Lee, B., Kim, P., Shin, B.S. and Choi, J., 2022. Comparison of the exposure assessment of di (2-ethylhexyl) phthalate between the PBPK model-based reverse dosimetry and scenario-based analysis: A Korean general population study. *Chemosphere*, p.133549.

Deierlein, A.L., Wu, H., Just, A.C., Kupsco, A.J., Braun, J.M., Oken, E., Soria-Contreras, D.C., Cantoral, A., Pizano, M.L., McRae, N. and Téllez-Rojo, M.M., 2022. Prenatal phthalates, gestational weight gain, and long-term weight changes among Mexican women. *Environmental Research*, p.112835.
