# Supplementary material for: Evaluating different web applications to assess the toxicity of plasticizers
Source: Sci Rep. 2022 Nov 16;12:19684. doi: 10.1038/s41598-022-18327-0 (PMC9668977; doi:10.1038/s41598-022-18327-0)
Supplement: Supplementary file 3 — Supplementary Figures. [file 41598_2022_18327_MOESM3_ESM.docx]

| 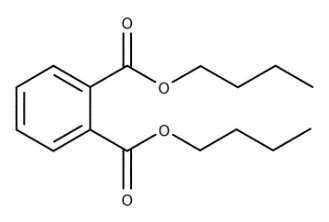 | **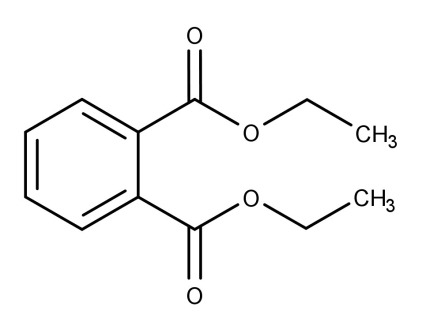** | | 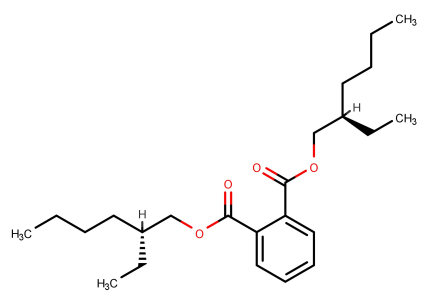 |
| --- | --- | --- | --- |
| [**DBP**](http://www.chemspider.com/Chemical-Structure.13837319.html?rid=f5eade38-4bf2-4150-9083-495063249abb) | [**DEP**](http://www.chemspider.com/Chemical-Structure.13837303.html?rid=2db4c6e9-bb76-43d4-86fb-29b9273bbaf0) | | [**DEHP**](http://www.chemspider.com/Chemical-Structure.5414319.html?rid=9730b3de-4d45-419a-b4a0-6bc10cd83083) |
| **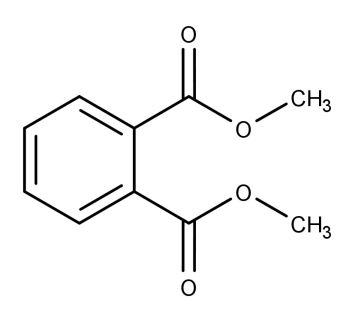** | **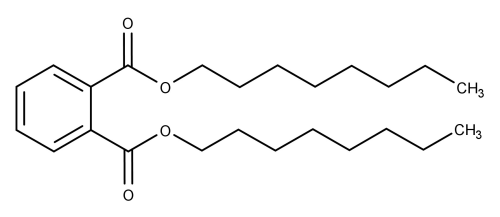** | | **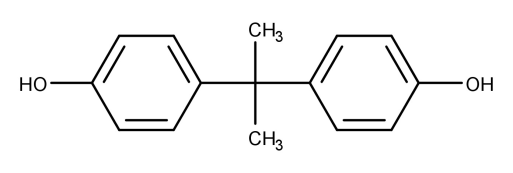** |
| [**DMP**](http://www.chemspider.com/Chemical-Structure.13837329.html?rid=8ff8b746-5103-46e8-8d0a-c2a679d61aba) | [**DNOP**](http://www.chemspider.com/Chemical-Structure.8043.html?rid=f715fe3d-57cb-4060-8994-8b10c152fed2) | | [**BPA**](http://www.chemspider.com/Chemical-Structure.6371.html?rid=bacefff6-ce79-4963-bfc7-c8ef089c109d) |
| **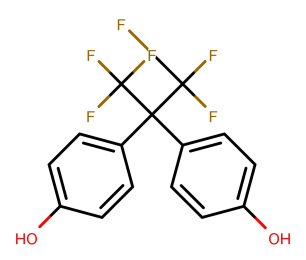** | **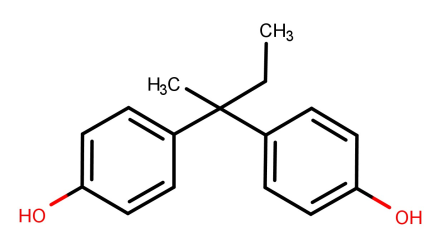** | | **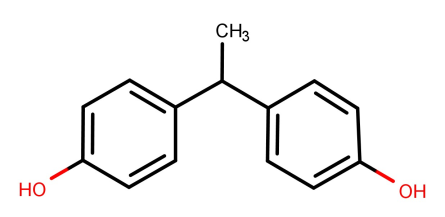** |
| [**BPAF**](http://www.chemspider.com/Chemical-Structure.66498.html?rid=f3a13617-a563-4d46-90d7-fc389c106d07) | [**BPB**](http://www.chemspider.com/Chemical-Structure.59553.html?rid=f32ddbd3-33dd-489f-9218-92484d1e40ed) | | [**BPE**](http://www.chemspider.com/Chemical-Structure.528599.html?rid=8ad47f17-125b-4063-b1d2-5692169afe75) |
| **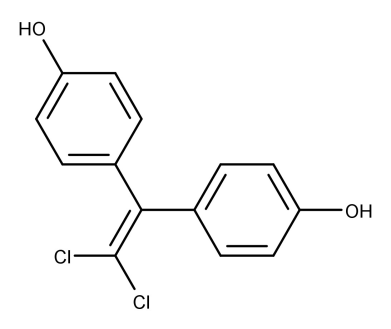** | **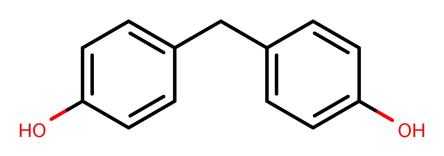** | | **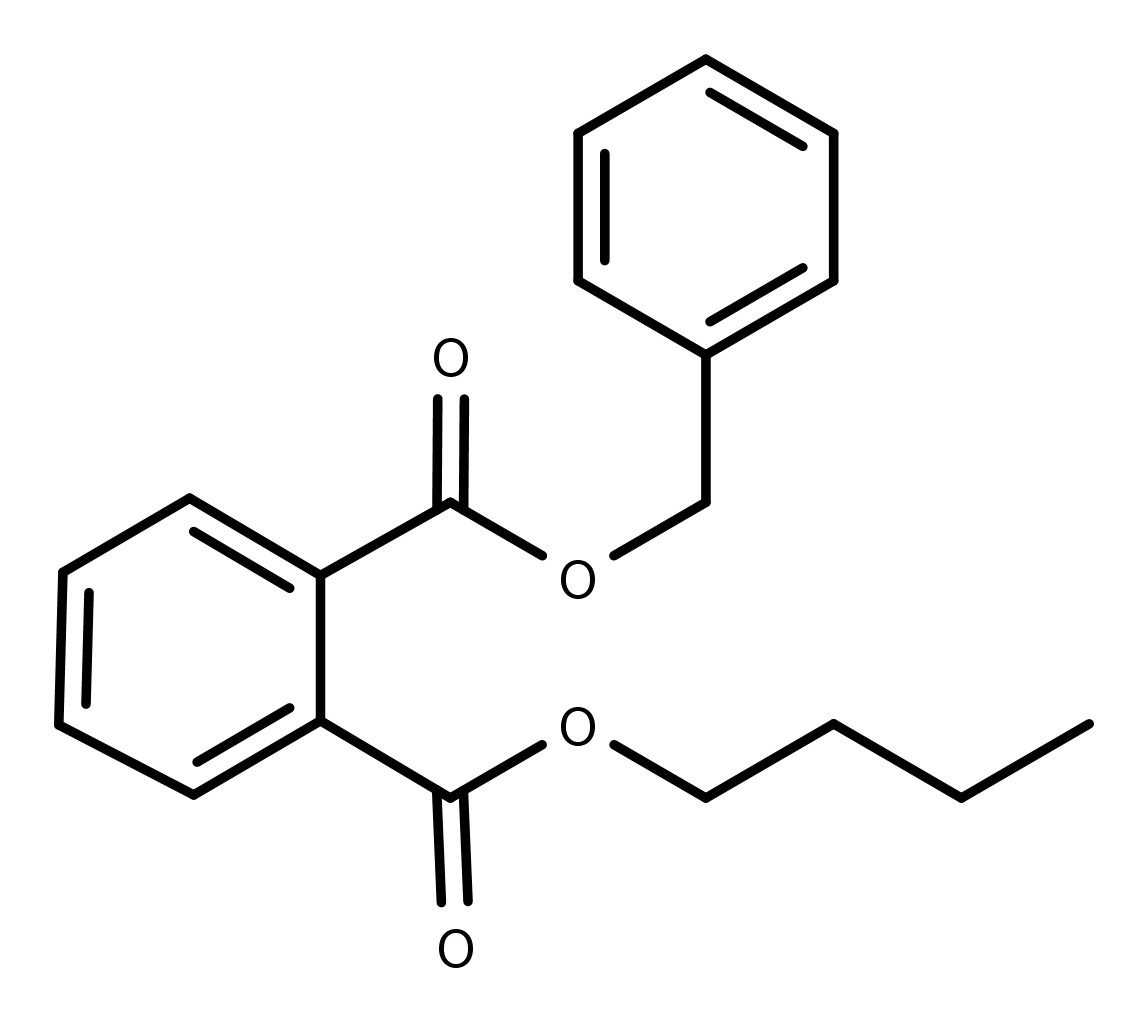** |
| [**BPC**](http://www.chemspider.com/Chemical-Structure.76387.html?rid=60aebec2-8214-4b5b-863f-c7ea9647feda) | [**BPF**](http://www.chemspider.com/Chemical-Structure.11614.html?rid=716a4e3d-bf53-4bb7-b300-9377f25ebe9b) | | [**BBP**](http://www.chemspider.com/Chemical-Structure.2257.html?rid=cf56bdb8-d7b5-49ac-8b36-8a9a4ae7444a) |
| **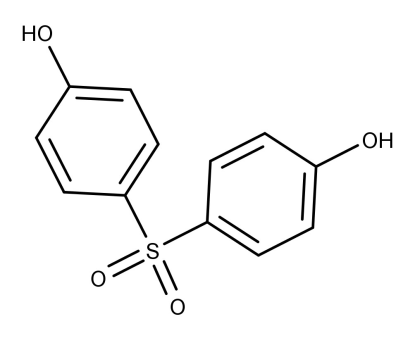** | | **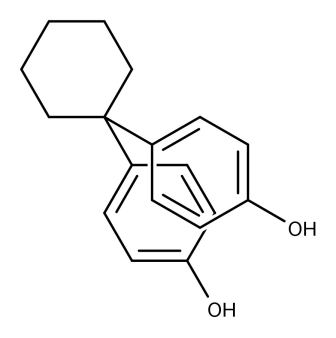** | |
| [**BPS**](http://www.chemspider.com/Chemical-Structure.6374.html?rid=b81b0d83-601e-428f-9222-9c80c4303744) | | [**BPZ**](http://www.chemspider.com/Chemical-Structure.202599.html?rid=c4c0f696-08d1-4322-ae2a-3938d79e9f48) | |

**S. Fig. 1.** Chemical structures of plasticizer accessed from the web page www.chemdata.dev.

**
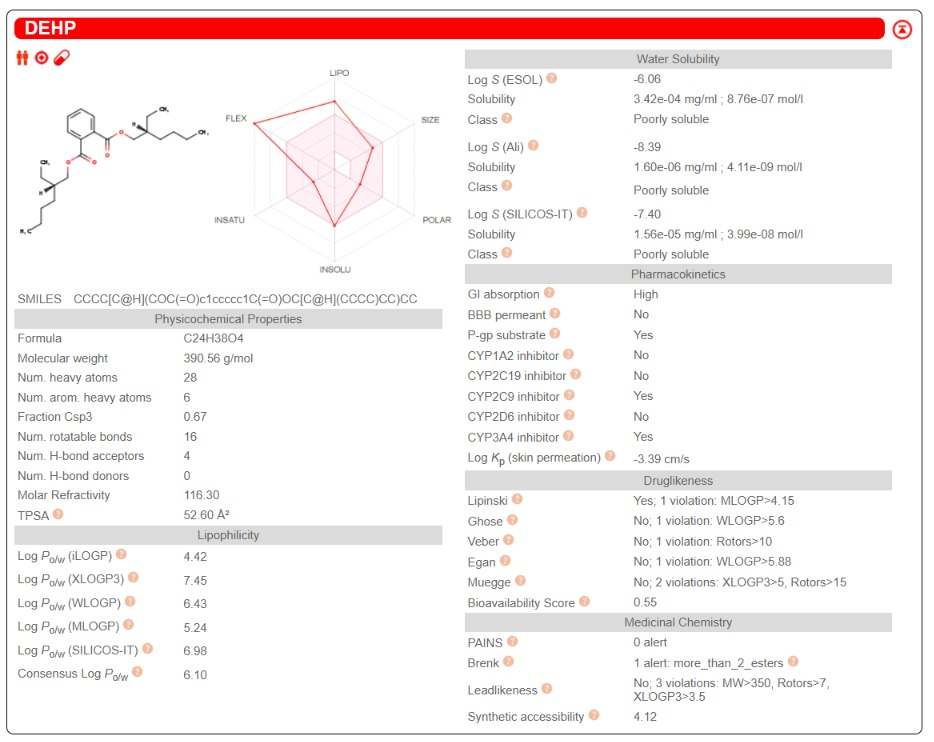
**

**S. Fig. 2.** Prediction of DEHP Physicochemical properties, Lipophilicity, water solubility, Pharmacokinetics, Druglikeness and Medicinal chemistry (www.swissadme.ch)
