# Supplementary material for: Evaluating different web applications to assess the toxicity of plasticizers
Source: Sci Rep. 2022 Nov 16;12:19684. doi: 10.1038/s41598-022-18327-0 (PMC9668977; doi:10.1038/s41598-022-18327-0)
Supplement: Supplementary file 4 — Supplementary Tables. [file 41598_2022_18327_MOESM4_ESM.docx]

**Supporting Material**

**S.Table 1.** Compound data from ChemSpider.

| Compound name | Abbreviations | Molecular Formula | Average mass  (da) | Monoisotopic mass  (da) | CSIDs* |
| --- | --- | --- | --- | --- | --- |
| benzyl butyl phthalate | BBP | C_19_H_20_O_4_ | 312.360 | 312.136169 | 2257 |
| dibutyl phthalate | DBP | C_16_H_22_O_4_ | 278.344 | 278.151794 | 13837319 |
| diethyl phthalate | DEP | C_12_H_14_O_4_ | 222.237 | 222.089203 | 13837303 |
| di-(2-ethylhexyl)phthalate | DEHP | C_24_H_38_O_4_ | 390.556 | 390.277008 | 5414319 |
| dimethyl phthalate | DMP | C_10_H_10_O_4_ | 194.184 | 194.057907 | 13837329 |
| dioctyl phthalate | DNOP | C_24_H_38_O_4_ | 390.556 | 390.277008 | 8043 |
| bisphenol A | BPA | C_15_H_16_O_2_ | 228.286 | 228.115036 | 6371 |
| bisphenol AF | BPAF | C_15_H_10_F_6_O_2_ | 336.229 | 336.058502 | 66498 |
| bisphenol B | BPB | C_16_H_18_O_2_ | 242.313 | 242.130676 | 59553 |
| bisphenol E | BPE | C_14_H_14_O_2_ | 214.260 | 214.099380 | 528599 |
| bisphenol C | BPC | C_14_H_10_Cl_2_O_2_ | 281.134 | 280.005798 | 76387 |
| bisphenol F | BPF | C_13_H_12_O_2_ | 200.233 | 200.083725 | 11614 |
| bisphenol S | BPS | C_12_H_10_O_4_S | 250.270 | 250.029984 | 6374 |
| bisphenol Z | BPZ | C_18_H_20_O_2_ | 268.350 | 268.146332 | 202599 |

**CSIDs - ChemSpider Identifier number.*

**S.Table 2.** SMILES of Plasticizers.

| Compound^a^ | SMILES |
| --- | --- |
| BBP | CCCCOC(=O)C1=CC=CC=C1C(=O)OCC1=CC=CC=C1 |
| DBP | CCCCOC(=O)C1=CC=CC=C1C(=O)OCCCC |
| DEP | CCOC(=O)C1=CC=CC=C1C(=O)OCC |
| DEHP | [H][C@](CC)(CCCC)COC(=O)C1=CC=CC=C1C(=O)OC[C@]([H])(CC)CCCC |
| DMP | COC(=O)C1=CC=CC=C1C(=O)OC |
| DNOP | CCCCCCCCOC(=O)C1=CC=CC=C1C(=O)OCCCCCCCC |
| BPA | CC(C)(C1=CC=C(O)C=C1)C1=CC=C(O)C=C1 |
| BPAF | OC1=CC=C(C=C1)C(C1=CC=C(O)C=C1)(C(F)(F)F)C(F)(F)F |
| BPB | CCC(C)(C1=CC=C(O)C=C1)C1=CC=C(O)C=C1 |
| BPC | OC1=CC=C(C=C1)C(=C(Cl)Cl)C1=CC=C(O)C=C1 |
| BPE | CC(C1=CC=C(O)C=C1)C1=CC=C(O)C=C1 |
| BPF | OC1=CC=C(CC2=CC=C(O)C=C2)C=C1 |
| BPS | OC1=CC=C(C=C1)S(=O)(=O)C1=CC=C(O)C=C1 |
| BPZ | OC1=CC=C(C=C1)C1(CCCCC1)C1=CC=C(O)C=C1 |

^a^ Abbreviations given in STable 1, *SMILES build by MarvinSketch 17.6. (https://chemaxon.com)*

**S.Table 3.** Mutagenicity of compounds as predicted by the LAZAR tool.

| Compound^a^ | Non-mutagenic  probability score | Mutagenic probability score | Mutagenicity prediction |
| --- | --- | --- | --- |
| BBP | 0.463 | 0.204 | NM |
| DBP | 0.685 | 0.224 | NM |
| DEP | 0.359 | 0.224 | NM |
| DEHP | 0.482 | 0.0809 | NM |
| DMP | 0.441 | 0.309 | NM |
| DNOP | 0.782 | 0.218 | NM |
| BPA | 0.264 | 0.236 | NM |
| BPAF | 0.266 | 0.234 | NM |
| BPB | 0.323 | 0.277 | NM |
| BPC | 0.232 | 0.223 | NM |
| BPE | 0.203 | 0.197 | NM |
| BPF | 0.221 | 0.223 | M |
| BPS | 0.215 | 0.185 | NM |
| BPZ | 0.27 | 0.23 | NM |

^a^ Abbreviations given in STable 1, ***M****=Mutagen,* ***NM****=Non-Mutagen.*

**S.Table 4.** Bee acute toxicity prediction by VEGA-KNN/IRFMN v.1.0.0.

| Compound^a^ | Bee acute toxicity | |
| --- | --- | --- |
|  | μg/bee | Toxicity class |
| BBP | over 100 | LT |
| DBP | lower than 1 | ST |
| DEP | between 1 and 100 | MT |
| DEHP | over 100 | LT |
| DMP | between 1 and 100 | MT |
| DNOP | over 100 | LT |
| BPA | over 100 | LT |
| BPAF | between 1 and 100 | MT |
| BPB | over 100 | LT |
| BPC | between 1 and 100 | MT |
| BPE | over 100 | LT |
| BPF | between 1 and 100 | MT |
| BPS | over 100 | LT |
| BPZ | over 100 | LT |

*^a^ Abbreviations given in STable 1,* ***ST****=Strong Toxicity,* ***MT****=Moderate Toxicity, and* ***LT****=Low Toxicity.*

**S.Table 5.** Fish acute toxicity prediction by VEGA-SarPy/IRFMN v.1.0.2.

| Compound^a^ | Fish Acute (LC50) mg/L | Toxicity class |
| --- | --- | --- |
| BBP | less than 1^R^ | 1 |
| DBP | less than 1 | 1 |
| DEP | between 10 and 100 | 3 |
| DEHP | less than 1 | 1 |
| DMP | between 10 and 100 | 3 |
| DNOP | less than 1 | 1 |
| BPA | between 10 and 100 | 3 |
| BPAF | between 10 and 100 | 3 |
| BPB | between 10 and 100 | 3 |
| BPC | between 10 and 100 | 3 |
| BPE | between 10 and 100 | 3 |
| BPF | between 10 and 100 | 3 |
| BPS | between 10 and 100 | 3 |
| BPZ | between 10 and 100 | 3 |

*^a^ Abbreviations given in STable 1, R=Reliable.*

**S.Table 6.** Rodent oral toxicity of compound predicted by PROTOX tool.

| Compound^a^ | LD50 mg/kg | Toxicity class |
| --- | --- | --- |
| BBP | 2330 | 5 |
| DBP | 3474 | 5 |
| DEP | 6172 | 6 |
| DEHP | 1340 | 4 |
| DMP | 1850 | 4 |
| DNOP | 1340 | 4 |
| BPA | 1000 | 4 |
| BPAF | 3400 | 5 |
| BPB | 1000 | 4 |
| BPC | 1770 | 4 |
| BPE | 1000 | 4 |
| BPF | 1000 | 4 |
| BPS | 1600 | 4 |
| BPZ | 1620 | 4 |

*^a^ Abbreviations given in STable 1.*

**S.Table 7.** Carcinogenicity of compound predicted by the CarcinoPred-EL tool.

| EOCs |  | Toxicity class |  |
| --- | --- | --- | --- |
|  | RF^a^ | SVM^a^ | XGBoost^a^ |
| BBP | C | NC | NC |
| DBP | C | NC | NC |
| DEP | NC | NC | NC |
| DEHP | C | NC | C |
| DMP | NC | NC | NC |
| DNOP | C | NC | C |
| BPA | NC | NC | NC |
| BPAF | NC | NC | NC |
| BPB | NC | NC | NC |
| BPC | NC | NC | NC |
| BPE | NC | NC | NC |
| BPF | NC | NC | NC |
| BPS | NC | NC | NC |
| BPZ | NC | NC | NC |

*^a^ Abbreviations given in STable 1, C=Carcinogen, NC=Non-Carcinogen.*
